# Tuning osteoporotic macrophage responses to favour regeneration by Cu-bearing titanium alloy in *Porphyromonas gingivalis* lipopolysaccharide-induced microenvironments

**DOI:** 10.1093/rb/rbaa045

**Published:** 2020-12-03

**Authors:** Xiongcheng Xu, Yanjin Lu, Ling Zhou, Mengjiao He, Jin Zhuo, Quan Zhong, Kai Luo, Jinxin Lin

**Affiliations:** 1 Fujian Key Laboratory of Oral Diseases & Fujian Provincial Engineering Research Center of Oral Biomaterial & Stomatological Key laboratory of Fujian College and University, School and Hospital of Stomatology, Fujian Medical University, Fuzhou 350002, China; 2 Institute of Stomatology & Laboratory of Oral Tissue Engineering, School and Hospital of Stomatology, Fujian Medical University, Fuzhou 350002, China; 3 Key Laboratory of Optoelectronic Materials Chemistry and Physics, Fujian Institute of Research on the Structure of Matter, Chinese Academy of Sciences, Fuzhou 350002, China; 4 University of Chinese Academy of Sciences, Beijing 1000049, China; 5 Department of Stomatology, Fujian Provincial Governmental Hospital & Fujian Health College Affiliated Hospital, Fuzhou 350003, China

**Keywords:** copper, osteoporosis, macrophages, inflammation

## Abstract

Guided bone regeneration in inflammatory microenvironments of osteoporotic patients with large alveolar bone defects remains a great challenge. Macrophages are necessary for alveolar bone regeneration *via* their polarization and paracrine actions. Our previous studies showed that Cu-bearing Ti6Al4V alloys are capable of regulating macrophage responses. When considering the complexity of oral microenvironments, the influences of Cu-bearing Ti6Al4V alloys on osteoporotic macrophages in infectious microenvironments are worthy of further investigations. In this study, we fabricated Ti6Al4V-Cu alloy by selective laser melting technology and used *Porphyromonas gingivalis* lipopolysaccharide (*P.g*-LPS) to imitate oral pathogenic bacterial infections. Then, we evaluated the impacts of Ti6Al4V-Cu on osteoporotic macrophages in infectious microenvironments. Our results indicated that Ti6Al4V-Cu not only inhibited the *P.g-*LPS-induced M1 polarization and pro-inflammatory cytokine production of osteoporotic macrophages but also shifted polarization towards the pro-regenerative M2 phenotype and remarkably promoted anti-inflammatory cytokine release. In addition, Ti6Al4V-Cu effectively promoted the activity of COMMD1 to potentially repress NF-κB-mediated transcription. It is concluded that the Cu-bearing Ti6Al4V alloy results in ameliorated osteoporotic macrophage responses to create a favourable microenvironment under infectious conditions, which holds promise to develop a GBR-barrier membrane for alveolar bone regeneration of osteoporosis patients.

## Introduction

Periodontitis, which is highly prevalent in the aging population, is a biofilm-induced inflammatory disease that is characterized by the destruction of the alveolar bone, periodontium and cementum. Due to the increasing number of individuals in the aging population, aging patients with periodontitis often suffer from other systemic diseases, such as osteoporosis [1]. The progression of periodontitis in osteoporotic patients develops quickly, which may result in tooth loss and easily cause severe alveolar bone defects [2]. The high degree of alveolar bone defect regeneration in these patients after tooth extraction for further dental implant placements currently deserves more attention.

Guided bone regeneration (GBR) has been verified to be a clinically reliable approach for restoring and regenerating alveolar bone *via* the placement of a GBR membrane as a barrier between the gingival tissues and the defects in order to provide a space to allow the incorporation of osteogenic cells and to prevent soft tissue cells from occupying the defects [3]. Titanium mesh, as an advantageous barrier membrane, has been applied for treating large alveolar bone defects due to its rigid structure and its space-making effect of maintaining volumetric stability without collapsing [4]. However, titanium mesh lacks adequate biological functions for the promotion of new tissue formation; therefore, the regeneration process requires a long-time duration, especially for osteoporotic patients. In addition, the sharp edges of the titanium mesh after manipulation and the extended tension in the tissue surrounding the wound may cause gingival and mucosal tissue dehiscence and may facilitate oral bacterial invasion, which can subsequently induce local inflammation; this situation may ultimately lead to alveolar bone regeneration failure [5].

As one of the essential trace elements, copper (Cu) serves a critical role in a series of metabolic processes [6]. Cu deficiency has been proven to be a potential pathogenic factor of osteoporosis, and Cu supplementation is recommended [6, 7]. In addition, the use of Cu in regenerative medicine has been proposed due to its broad-spectrum antibacterial activities and its stimulating effects on a variety of cellular activities [8–11]. Our previous studies have shown that Cu-bearing Ti6Al4V alloys can be used to fabricate patient–individual mesh to fit alveolar bone defects by the use of selective laser melting (SLM) technology. Importantly, the Ti6Al4V-Cu displayed brilliant antibacterial properties and biocompatibility. Thus, this alloy may overcome the limitations of titanium mesh for potential clinical GBR applications [12–14].

Macrophages, which are the indispensable players in innate immunity, act as key regulators in the onset and progression of bacteria-induced inflammation [15]. Cytokines and chemokines released from macrophages are in favour of the clearance of invading bacteria [16, 17]. However, it has been reported that inflammation-induced tissue damage is mainly attributed to the over-reaction of macrophages in response to bacterial infections instead of being attributed to direct damage by invading bacteria [18]. In addition, the dysfunction in bone metabolism is capable of exerting pro-inflammatory effects of osteoporotic macrophages in response to bacteria [19–21]. Over-reactions of osteoporotic macrophages to bacteria appear to be theoretically difficult to ameliorate. In addition, macrophages are necessary for alveolar bone and periodontal regeneration *via* their polarization, paracrine actions and cell interactions, which create a favourable microenvironment [17]. Our previous studies have indicated that Cu-bearing Ti6Al4V alloys can reduce inflammatory responses of macrophages [13, 14], which may be beneficial for alveolar bone regeneration. When considering the complexity of oral microenvironments, the effects of Cu-bearing Ti6Al4V alloy on osteoporotic macrophages in infectious microenvironments are worthy of further investigations.

*Porphyromonas gingivalis* (*P.g*), which is one of the best-characterized oral pathogenic bacteria, is present in the biofilms on teeth and is closely associated with periodontitis [22]. Lipopolysaccharide (LPS), which is the main virulence factor produced by *P.g*, frequently induces uncontrolled inflammatory responses *via* the activation of innate immune responses [23]. *P.g*-LPS ligates toll-like receptor-2 (TLR-2) and can either evade or antagonize toll-like receptor-4 (TLR-4) activation in macrophages, unlike LPS from other bacteria (such as *Escherichia coli*), which binds to TLR-4 to induce inflammatory disorders, including increased M1 polarization and pro-inflammatory cytokine expression [24]. Thus, we used *P.g*-LPS in this study to imitate oral pathogenic bacteria-induced surgical site infections. Then, we verified the hypothesis that the Cu-bearing Ti6Al4V alloy can alleviate the inflammatory responses of osteoporotic macrophages in *P.g*-LPS-induced infectious microenvironments to obtain a more sophisticated understanding of the anti-infectious and pro-regenerative properties of this alloy and to provide further evidence for the clinical application of this novel biomaterial.

## Materials and methods

### Sample preparation

In our previous study, a group of Cu-bearing Ti6Al4V-*x*Cu (*x* = 0, 2, 4 and 6 wt%) alloys were fabricated by SLM technology and Ti6Al4V-6Cu displayed brilliant properties [12]. Here, the Ti6Al4V-Cu alloy (composed of 6% Cu) was fabricated by SLM technology as previously described. Ti6Al4V alloy fabricated in the same processing method was used as a control. Both alloys measuring 10 × 10 × 3 mm were used. All samples were ground and polished with SiC papers with 360, 1000 and 2000 grit sizes and SiO_2_–H_2_O_2_ solution before co-cultured with cells. Then they were cleaned with acetone, ethanol and distilled water, respectively, *via* ultrasonication.

**Figure 1.**
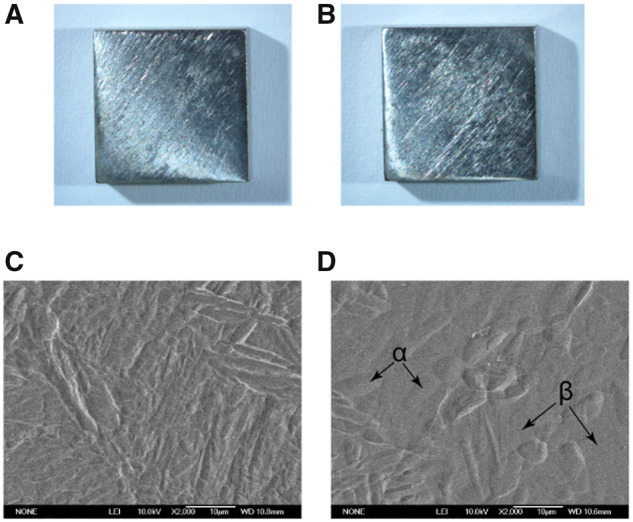
Surface images of the Ti6Al4V-Cu and Ti6Al4V alloys. Macroscopic views of Ti6Al4V (**A**) and Ti6Al4V-Cu (**B**). SEM images of Ti6Al4V (**C**) and Ti6Al4V-Cu (**D**).

For the microstructure observations, both the Ti6Al4V and the Ti6Al4V-Cu were etched in Kroll reagent (containing 50 ml distilled water, 5 ml HNO_3_ and 2 ml HF) *via* a standard metallographic procedure and were imaged using a scanning electron microscope (Phenom G2). Figure 1C and D shows the representative surface images of the Ti6Al4V-Cu and Ti6Al4V.

Prior to performing immersion test, Ti6Al4V−Cu were ground with SiC paper and immersed in phosphate-buffered saline (PBS) with a surface-area-to-volume ratio of 3 cm^2^/ml at 37°C in a humidified atmosphere containing 5% CO_2_ after 1, 3, 5 and 7 days to prepare the extracts. The amount of Cu ions was measured using an inductively coupled plasma-atomic emission spectroscopy (ICP-AES, Ultima2). Data are shown in Supplementary Fig. S.1.

### Culture of macrophages from osteoporotic rats

All procedures involving rat were approved by the Animal Care and Use Committee of Fujian Medical University. An osteoporosis rat model was established as described in our previous research [25]. Fifteen rats were ovariectomized bilaterally (OVX group). An equal volume of fat tissue was removed from a location next to each ovary of five rats (Sham group). Rat weight of each group was measured. After 12 weeks, rat tibiae were harvested to fixed in paraformaldehyde, decalcified in an EDTA solution and sectioned to stain with Hematoxylin and Eosin (H&E) to identify osteoporosis of rats.

Osteoporotic macrophages were cultured from femur bone marrow of OVX group rat as described previously [14]. The characterization of osteoporotic macrophages was performed with the standard surface markers by flow cytometry. Macrophages were suspended in PBS-containing fluorescein isothiocyanate (FITC)-coupled antibodies against CD11b or an isotype control, FITC-coupled non-specific IgG (Invitrogen, USA).

Osteoporotic macrophages at a density of 1 × 105 cells per well were seeded on both alloys in 24-well culture plates. In brief, 10 μg/ml of *P.g-*LPS (Invivogen, USA) was added to the medium to induce the infectious microenvironments as described previously [23, 24, 26].

### Cell morphology and immunofluorescence staining

To observe cells on both alloys, they were washed with PBS and fixed with paraformaldehyde. The cells were incubated with primary antibody against CCR7 and MRC1 (Abcam, USA). Then the cells were washed with PBS and incubated with Alexa-Fluor 488 secondary antibodies (Invitrogen, USA). Cytoskeleton of osteoporotic macrophages was stained with rhodamine phalloidin (Cytoskeleton Inc., USA). Cell nuclei were stained by DAPI solution (Beyotime, China) and were observed using a fluorescence microscopy (Olympus, Japan).

Osteoporotic macrophage perimeter and spread area on both alloys were assessed using ImageJ software (National Institutes of Health, USA). Then the circularity was analysed. Cell Circularity = (4π × area)/(perimeter)^2^. A perfect circle is equals to 1. As the shape becomes more convoluted, the value decreases.

### Quantitative real-time PCR analysis

RNA of macrophages cultured on both alloys was extracted using TRIzol reagent (Life Technologies, USA). The RNA was transcribed using a PrimeScript RT reagent kit (Takara, Japan). cDNA amplification and detection were performed using a Roche LightCycler480 real-time PCR system with a SYBR green PCR reaction mix (Takara, Japan). All primers used in the experiments are listed in Table 1. The relative gene expression level was normalized to the internal control (*GAPDH*), based on the 2^−ΔΔCt^ method.

**Table 1.** Primer sequences used for quantitative real-time PCR

Gene	Forward primer sequence (5′-3′)	Reverse primer sequence (5′-3′)
*GAPDH*	CGGCAAGTTCAACGGCACAGTCAAGG	ACGACATACTCAGCACCAGCATCACC
*iNOS*	AGAGACGCACAGGCAGAG	AGGCACACGCAATGATGG
*CCR7*	TACCTGGTTATCATCCGCACTC	TGGAAGACGACGAACACTACG
*MRC1*	GTTGACTGTGTTGTTGTGATTGG	GCCGTGGTTGGAGAGATAGG
*ARG1*	GGCAGTGGCGTTGACCTTG	TGGGAGGAGCAGCGTTGG
*IL-1β*	GTTTCCCTCCCTGCCTCTGAC	GACAATGCTGCCTCGTGACC
*IL-6*	ATGGAGGAGGCACAGTCAGATG	AACCTAAGCAAGCGAGCAAGC
*IL-10*	CACCCACTTCCCAGTCAGC	AATCTGTCAGCAGTATGTTGTCC
*TNF-α*	TGGCGTGTTCATCCGTTCTCTAC	CTACTTCAGCGTCTCGTGTGTTTC
*COMMD1*	CACGGCACTCAACTCAAATAC	CAAACTCCAGACACAGAAACTC
*NF-κB*	TATGGCTTCCCGCACTATGG	CTCCCTGTCGTCACTCTTGG

### Enzyme-linked immunosorbent assay

Inflammatory cytokine secretions of macrophages on both alloys were measured using an enzyme-linked immunosorbent assay (ELISA) kit (Abcam, Cambridge, USA). The supernatant was collected and quantified. The amount of IL-1β, IL-6, IL-10 or TNF-α per milliliter of supernatant was determined by correlation with a standard curve.

### Western blotting

Cells cultured on both alloys were lysed in RIPA containing 1 mM containing Phenylmethanesulfonyl fluoride. Proteins were separated on an SDS-PAGE gel, transferred onto polyvinylidene difluoride membranes (Millipore, Billerica, USA) and blocked. The blotted membranes were incubated with primary antibodies specific for CCR7 (Abcam, USA), MRC1 (Abcam, USA) and GAPDH (Boster, China), and then incubated with secondary antibodies (Boster, China). The protein bands were detected using an ECL detection kit (Millipore, USA) and were acquired with the Chemiluminescence Imaging System (CLINX, China).

### Statistical analysis

All of the data shown represent the mean ± SD. Statistical analysis was performed using Student’s *t*-test or one-way ANOVA followed by Tukey’s HSD *post-hoc* test. The differences were considered significant at *P *<* *0.05.

## Results

### Establishment of the osteoporotic rat model and culture of osteoporotic macrophages

**Figure 2.**
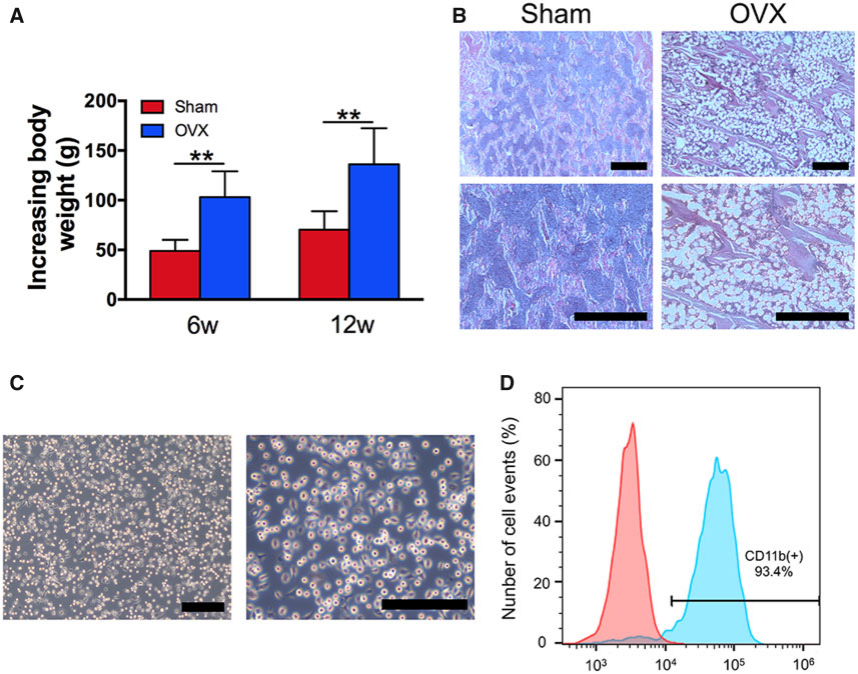
Establishment of osteoporosis rat model and culture of osteoporotic macrophages. (**A**) Increasing body weights of the Sham group and OVX group rats. (**B**) H&E-stained sections of tibias. (**C**) Morphological observations of osteoporotic macrophages. (**D**) Flow cytometric analysis of osteoporotic macrophages. Blue histogram represents the proportion of CD11b^+^ cells. Red histogram represents isotype controls. **P <* *0.01. Bars = 2 μm

After ovariectomy or fat tissue-excising surgery, the increases in the body weights of the sham group and the OVX group were measured and are shown in Fig. 2A. The increased body weights of the OVX group rats were significantly higher than those of the sham group rats after 6 and 12 weeks of surgery (Fig. 2A). To further identify rat osteoporosis, the tibias of the rats were resected and histologically processed to obtain H&E-stained sections. Compared with compact structures of the tibias in the sham group rats, those of the OVX group rats exhibited expansions of the bone marrow area, including looser constructions of more widely separated trabeculae (Fig. 2B). All of these results indicated that the osteoporosis rat model was successfully established *via* ovariectomy.

Bone marrow-derived macrophages were isolated from osteoporotic rats *in vitro*. The cell morphology was observed by the use of an inverted microscope. As shown in Fig. 2C, bone marrow-derived macrophages appeared round, spindle-like or polygonal, and some of the macrophages contained few pseudopodia. Osteoporotic macrophages were characterized by the expression of CD11b, which is a characteristic of the macrophage lineage. The rates of CD11b^+^ expression on bone marrow-derived macrophages were approximately 93.4% (Fig. 2D). Taken together, the osteoporotic macrophages were successfully cultured from osteoporosis rats.

### Morphological features and quantification

**Figure 3.**
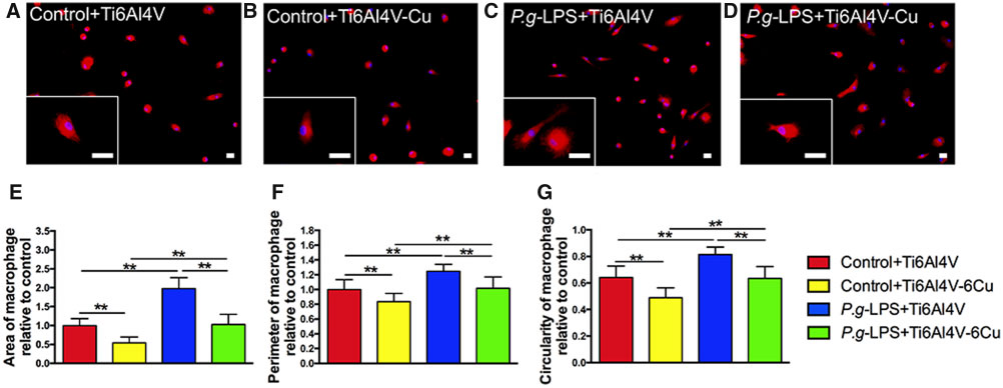
Morphological features and quantification of osteoporotic macrophages on Ti6Al4V and Ti6Al4V-Cu under *P.g-*LPS stimulation. Representative images of the magnified views of macrophages are shown (**A–D**). Statistical results of the macrophage spreading area (**E**), the perimeter (**F**) and the circularity (**G**). Immunofluorescence staining of phalloidin (red) and DAPI (blue) in macrophages. Bars = 2 μm. *P <* *0.05 and **P <* *0.01

Figure 3 shows the osteoporotic macrophages on the surfaces of the Ti6Al4V and Ti6Al4V-Cu alloys (both with and without the presence of *P.g-*LPS) through immunofluorescence staining. The osteoporotic macrophages on Ti6Al4V-Cu exhibited more elongated and spindle-like morphologies (Fig. 3B), which are recognized as characteristic features of M2 macrophages. Osteoporotic macrophages on Ti6Al4V were more rounded with apparent pseudopodia (Fig. 3A), compared to those cultured on Ti6Al4V. Compared to the control groups, osteoporotic macrophages on both alloys exhibited more pseudopodia under *P.g-*LPS stimulation. In addition, osteoporotic macrophages exhibited a more fried egg-shaped morphology in the *P.g-*LPS + Ti6Al4V group (Fig. 3C), which is a typical feature of M1 macrophages, than those in the *P.g-*LPS + Ti6Al4V-Cu group (Fig. 3D).

The morphological quantification was performed using ImageJ software. The macrophage spreading area and the perimeter of the osteoporotic macrophages in response to *P.g-*LPS stimulation significantly increased (Fig. 3E and F) when compared to the control groups, indicating that macrophages were activated. A decreased cell spreading area and smaller perimeter were observed for those cells cultured on Ti6Al4V-Cu, compared to Ti6Al4V. The circularity of the macrophages defines the degree of polarization. Figure 2G shows that Ti6Al4V-Cu decreased circularity of the osteoporotic macrophages in the presence of *P.g*-LPS. These results indicated that Ti6Al4V-Cu inhibited the activation of osteoporotic macrophages under *P.g*-LPS stimulation at the morphological level.

### Polarization-related gene expression

**Figure 4.**
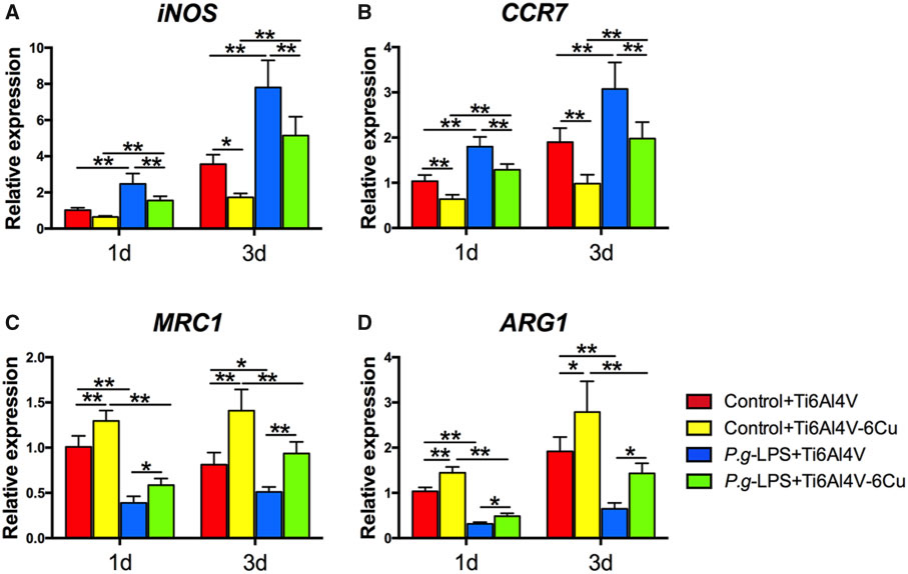
Polarization-related gene expression of osteoporotic macrophages on Ti6Al4V and Ti6Al4V-Cu under *P.g-*LPS stimulation. (**A**) *iNOS*, (**B**) *CCR7*, (**C**) *MRC1* and (**D**) *ARG1*. *P <* *0.05 and **P <* *0.01

The impacts of Ti6Al4V-Cu on osteoporotic macrophages were evaluated under *P.g*-LPS stimulation. The expression of polarization-related genes was assessed by quantitative real-time PCR. Increased expression of M1 macrophage polarization marker genes (*iNOS* and *CCR7*) was detected on the surface of Ti6Al4V (Fig. 4A and B). Osteoporotic macrophages on Ti6Al4V-Cu expressed lower levels of M1 macrophage marker genes than those on Ti6Al4V under *P.g-*LPS stimulation. M2 macrophage polarization marker gene (*MRC1* and *ARG1*) expression levels of osteoporotic macrophages that were stimulated with *P.g-*LPS were observed to decrease significantly (Fig. 4C and D). Ti6Al4V-Cu promoted the expression levels of M2 macrophage marker genes in the osteoporotic macrophages both with and without the presence of *P.g-*LPS, in comparison to Ti6Al4V, which suggested that Ti6Al4V-Cu increased M2 macrophage marker gene expression of osteoporotic macrophages that were stimulated with *P.g-*LPS.

### Polarization-related protein expression

**Figure 5.**
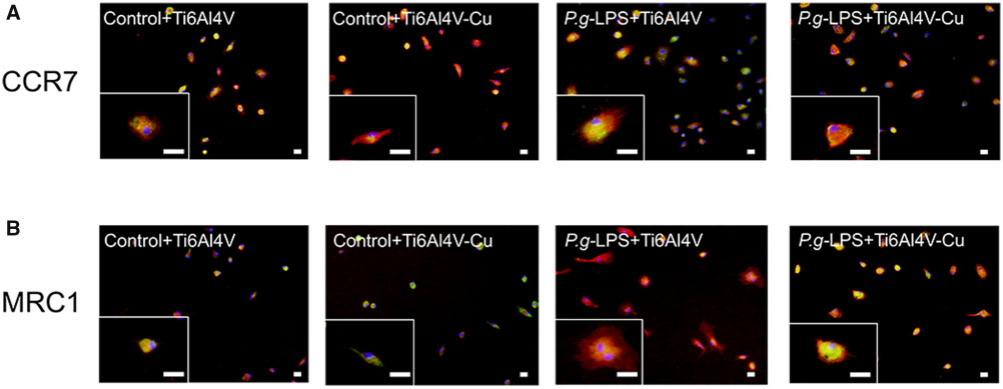
Immunofluorescence staining of polarization-related markers in osteoporotic macrophages on Ti6Al4V and Ti6Al4V-Cu in response to *P.g-*LPS stimulation. Representative images of magnified views of macrophages are also shown. CCR7 (**A**) and MRC1 (**B**) were stained green by the use of Alexa Fluor 488-conjugated secondary antibodies, cytoskeletons were stained red with phalloidin and the nuclei were stained blue with DAPI. Bars = 2 μm

Polarization-related protein expression was detected by immunofluorescence microscopy. Fluorescent images are shown in Fig. 5. Figure 5A shows a remarkable increase in the expression of CCR7 of osteoporotic macrophages that were stimulated with *P.g-*LPS, and the expression levels of macrophages on Ti6Al4V-Cu decreased. MRC1 expression of the osteoporotic macrophages decreased after stimulation with *P.g-*LPS, and Ti6Al4V-Cu promoted the expression of MRC1 compared to those cells that were cultured on Ti6Al4V (Fig. 5B).

**Figure 6.**
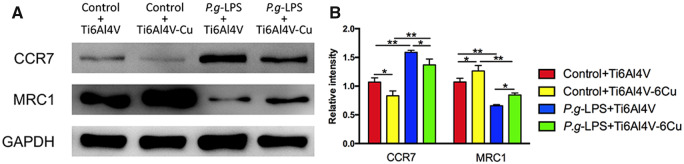
Polarization-related protein expression of osteoporotic macrophages on Ti6Al4V and Ti6Al4V-Cu under *P.g-*LPS stimulation. CCR7 and MRC1 protein expression levels were analysed and quantified by Western blotting. *P <* *0.05 and **P <* *0.01

Western blotting analysis was performed to verify the effects of Ti6Al4V-Cu on osteoporotic macrophages in response to *P.g-*LPS stimulation (Fig. 6A and B). The results were consistent with the quantitative real-time PCR analyses and with the immunofluorescence staining observations. Ti6Al4V-Cu reduced the CCR7 expression levels and promoted the MRC1 expression levels of osteoporotic macrophages that were stimulated with *P.g-*LPS compared to those on Ti6Al4V. Taken together, Ti6Al4V-Cu accelerated the M2 marker expression of osteoporotic macrophages in response to *P.g-*LPS stimulation.

### Inflammatory cytokine release

**Figure 7.**
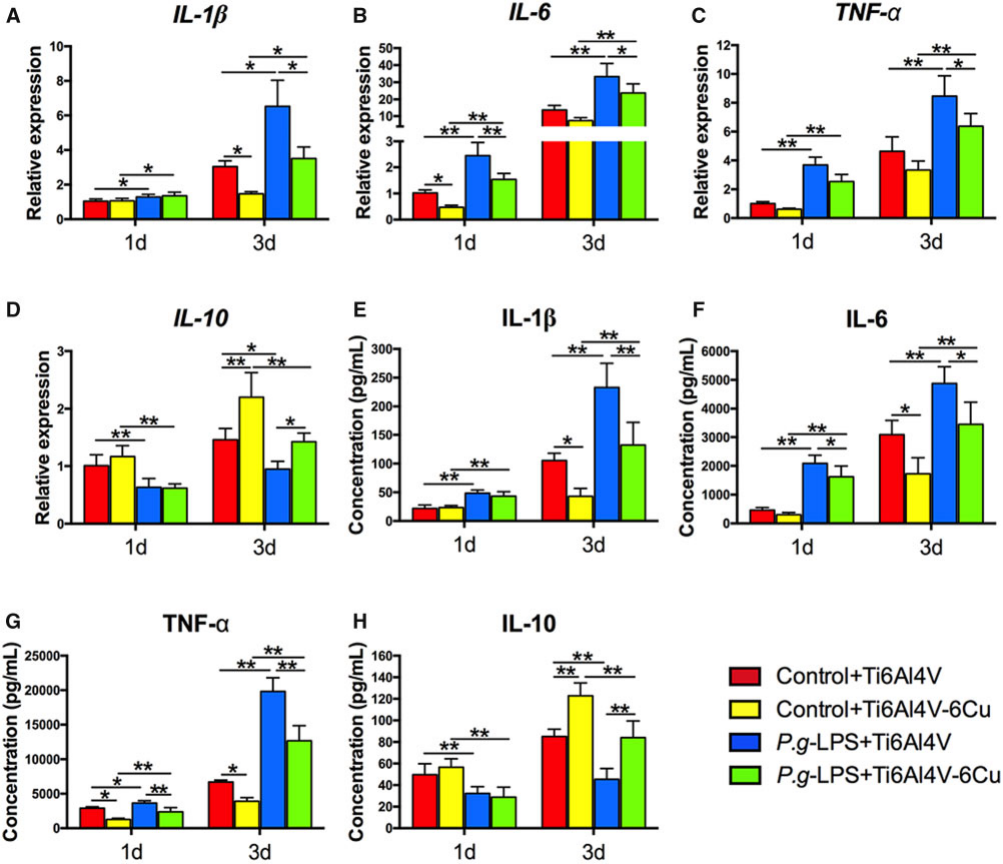
Inflammatory cytokine expression of osteoporotic macrophages on Ti6Al4V and Ti6Al4V-Cu under *P.g-*LPS stimulation. Inflammatory gene expression of *IL-1β* (**A**), *IL-6* (**B**), *TNF-α* (**C**) and *IL-10* (**D**). Inflammatory protein expression of IL-1β (**E**), IL-6 (**F**), TNF-α (**G**) and IL-10 (**H**). *P <* *0.05 and **P <* *0.01

The inflammatory cytokine gene expression of osteoporotic macrophages was assessed using quantitative real-time PCR. The pro-inflammatory cytokine gene (*IL-1β*, *IL-6* and *TNF-α*) expression levels increased and the anti-inflammatory cytokine gene (*IL-10*) expression levels decreased under *P.g-*LPS stimulation (Fig. 7A–D). Osteoporotic macrophages that were stimulated with *P.g-*LPS on Ti6Al4V-Cu expressed lower levels of pro-inflammatory cytokine genes (*IL-1β*, *IL-6* and *TNF-α*) and higher levels of an anti-inflammatory cytokine gene (*IL-10*), in comparison to those on Ti6Al4V.

As shown in Fig. 7E–H, the inflammatory cytokine release of osteoporotic macrophages on both alloys in response to *P.g-*LPS stimulation was detected using ELISA. The addition of *P.g-*LPS led to a significant increase in pro-inflammatory cytokines (IL-1β, IL-6 and TNF-α) (Fig. 7E–G) and to a significant decrease in an anti-inflammatory cytokine (IL-10) (Fig. 7H) after 1 day and 3 days of culture. When osteoporotic macrophages were cultured on Ti6Al4V-Cu with *P.g-*LPS stimulation, a general decrease in the expression levels of IL-1β, IL-6 and TNF-α was observed compared to those macrophages that were cultured on Ti6Al4V, and IL-10 expression was increased. The results suggested that Ti6Al4V-Cu could modulate inflammatory cytokine release of osteoporotic macrophages under *P.g*-LPS stimulation.

### COMMD1 and NF-κB expression

**Figure 8.**
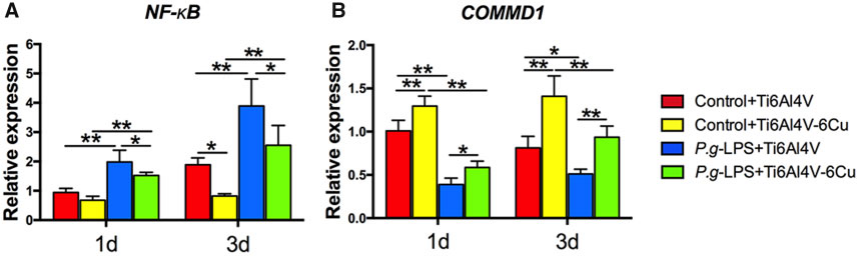
*COMMD1* and *NF-κB* expression of osteoporotic macrophages on Ti6Al4V and Ti6Al4V-Cu in response to *P.g-*LPS stimulation. *NF-κB* (**A**) and *COMMD1* (**B**) gene expression was analysed and quantified by quantitative real-time PCR. *P <* *0.05 and **P <* *0.01.

To further explore the potential anti-inflammation mechanism of osteoporotic macrophages that were stimulated with *P.g-*LPS on Ti6Al4V-Cu, we evaluated the expression of COMMD1 and NF-κB (Fig. 8). NF-κB expression of osteoporotic macrophages dramatically increased in response to *P.g-*LPS stimulation; however, NF-κB expression significantly decreased on Ti6Al4V-Cu (Fig. 8A). Furthermore, Ti6Al4V-Cu increased COMMD1 gene expression in osteoporotic macrophages in response to *P.g-*LPS stimulation (Fig. 8B) in comparison to Ti6Al4V. In short, Ti6Al4V-Cu may inhibit inflammatory responses of osteoporotic macrophages, presumably by up-regulating COMMD1 expression to down-regulate NF-κB expression during *P.g*-LPS stimulation.

## Discussion

Cu has been shown to be a multi-functional agent that not only increases angiogenesis and osteogenesis, but also inhibits osteoclastogenesis and displays broad-spectrum antibacterial properties [9, 11]. Numerous researches have demonstrated that Cu incorporated into a variety of biomaterials would promote their bioactivities [8–10]. Cu ions released from Cu-containing materials are involved in regulating the cellular activities and inhibiting growth of bacteria. The previous studies indicated that the addition of Cu in Ti6Al4V alloys would decrease inflammatory responses, and enhance blood vessel formation and bone regeneration due to the sustained release of Cu ions [11–14]. When considering the complexity of oral microenvironments, further investigation of Cu-bearing Ti6Al4V alloy on osteoporotic macrophages in infectious microenvironments is required.

Macrophages are activated at baseline to exert essentially protective effects in the clearance of bacterial pathogens during the early phase of infection. If the bacteria continue to grow, the macrophages can over-react to the bacterial challenge, leading to the release of inflammatory cytokines and the induction of self-tissue damage [18, 27]; this is especially true in susceptible osteoporotic patients. It has been reported that osteoporotic patients are susceptible to bacterial infections due to their higher degrees of inflammatory states [2]. Thus, there may be two ways to impede the influence of bacterial infections to optimize the regenerative microenvironment: by precluding the bacteria around the surgical site or by preventing the macrophage over-reaction to bacteria. *P.g*, one of the best-characterized oral pathogenic bacteria, is a Gram-negative anaerobe and grows in anaerobic condition. It is difficult to directly co-culture *P.g* and macrophages on the surface of Cu-bearing Ti6Al4V alloy *in vitro*. Thus, we imitated infectious microenvironments in this study through *P.g*-LPS stimulation. It has been proven that *P.g*-LPS would ligate TLR-2 to activate NF-κB signaling pathway, then induces a series of inflammatory responses, such as M1 macrophage polarization and pro-inflammatory cytokine secretion [23, 24]. Subsequently, Cu-bearing Ti6Al4V alloy, which was fabricated by SLM, was co-cultured with osteoporotic macrophages in *P.g*-LPS-induced infectious microenvironments. Our results indicate that the Cu-bearing Ti6Al4V alloys are able to impede macrophage over-reactions to bacterial pathogens and are also able to provide a microenvironment-favouring regeneration.

Macrophages are key regulators of the inflammation and tissue regeneration processes that play different roles depending on their subtypes [15]. The diversity and plasticity of macrophages enable them to perform certain functions, such as polarization and inflammatory cytokine secretion [17, 18]. In response to a bacterial challenge, macrophages undergo a re-programming that results in the emergence of pro-inflammatory M1 macrophage polarization and the production of inflammatory cytokines [28]. In addition, M2 macrophages would then be correspondingly activated at later phases of regeneration [28]. The dysfunction in bone metabolism may also alter the inflammatory states and immune activities of osteoporotic macrophages in response to bacteria [19–21]. The previous researches have indicated that the addition of Cu would result in a significant decrease in inflammatory responses in RAW264.7 cells [13, 29]. However, the macrophages in these previous studies were leukemia virus-transformed cell line-derived macrophages from BALB/c mice, which may not have fully exhibited physiological functions [30, 31]. In this study, macrophages were cultured from osteoporosis rats, which are able to exhibit more clinically relevant functions. Osteoporotic macrophages have also been observed to be more susceptible to bacterial infections [20, 21]. Accordingly, the responses of these macrophages to bacteria appear to be theoretically difficult to prevent. It has been reported that *P.g*-LPS can result in macrophage activation to induce a series of inflammatory responses [23]. However, to the best of our knowledge, no studies have focused on the influences of *P.g*-LPS on osteoporotic macrophages. This study indicated that the inflammatory responses of osteoporotic macrophages can dramatically increase and that there was a greater tendency towards M1 polarization, which occurred after *P.g*-LPS stimulation. Furthermore, the application of Cu-bearing Ti6Al4V alloy alleviated the inflammatory responses of osteoporotic macrophages in *P.g*-LPS-induced infectious microenvironments. Insights into the effects of Cu-bearing Ti6Al4V alloy on *P.g-*LPS-activated osteoporotic macrophages were evident from the cell polarization and cytokine secretion of macrophages. Therefore, it would appear that the Cu-bearing Ti6Al4V alloy can prevent excessive osteoporotic macrophage activation and the recruitment of macrophages to the site of infection during bacterial invasion, which is another method of preventing infection-induced tissue destruction *via* the inhibition of macrophage over-reaction. Interestingly, our results demonstrated that Cu-bearing Ti6Al4V alloy not only inhibited the *P.g-*LPS-induced M1 macrophage polarization and pro-inflammatory cytokine production of osteoporotic macrophages but also shifted polarization towards pro-regenerative M2 macrophages and promoted anti-inflammatory cytokine release during *P.g*-LPS stimulation. M2 macrophages have been proven to serve a predominant role in tissue regeneration through the release of anti-inflammatory and wound-healing cytokines to permit regeneration [28]. These characteristics may also define the anti-infectious properties of Cu-bearing Ti6Al4V alloy from another perspective.

Current studies have indicated that the biological properties of Cu-containing alloys are mainly associated with Cu ions [32, 33]. The contact of these alloys with cells is also related to biological functions. As an essential trace element, Cu is indispensable to the activities of a variety of enzymes, such as COMMD1 [34]. Cu is a catalytic cofactor for COMMD1, which acts as a suppressor of NF-κB by binding to a conserved domain that is present in all NF-κB subunits [34–36]. Canonical NF-κB activation triggers a signaling cascade that leads to the transcription of hundreds of genes that are involved in inflammation and immune homeostasis [37]. In light of its critical role in regulating inflammation, NF-κB activation in macrophages has been considered as a main factor. In this study, the NF-κB expression of osteoporotic macrophages dramatically increased during *P.g*-LPS stimulation, and this expression subsequently returned to a lower level on the surface of Cu-bearing Ti6Al4V alloys. In addition, the expression of COMMD1 correspondingly changed. Furthermore, several researchers have also observed that Cu-containing materials can inhibit endogenous NF-κB activation [38, 39]. However, another study indicated that Cu could activate the NF-κB pathway after a bolus injection of a large amount of Cu-containing agent [40]. When comparing and contrasting the differences among these studies, we infer that the wide disparity in the Cu content may account for the antagonistic effects. Our results (Supplementary Fig. S.1) indicate that Cu ions that are released from Ti6Al4V-Cu were within acceptable amounts. Therefore, we speculate that the inhibitory effect of Cu-bearing Ti6Al4V alloy on the over-reaction of osteoporotic macrophages might be attributed to Cu ions from the alloy, which can effectively promote the activity of COMMD1 to potentially repress NF-κB-mediated transcription.

Although the effects of Cu-bearing Ti6Al4V alloy on osteoporotic macrophages in infectious microenvironments have been extensively investigated, the specific mechanisms of the influences of Cu-bearing Ti6Al4V-mediated functions on the whole immune system remain unknown. We also need to identify the effects of Cu-bearing Ti6Al4V alloys through *in vitro* and *in vivo* studies. In addition, a new culture system is expected to explore macrophage-induced multicellular interactions for tissue regeneration. In addition, only *P.g*-LPS was used in this study to imitate oral pathogenic bacteria-induced infections. When considering the diversity of the oral microbiome, the effects of Cu-bearing Ti6Al4V alloy on a model of the inflammatory oral microbiome are worth verifying in future studies.

## Conclusion

**Figure 9.**
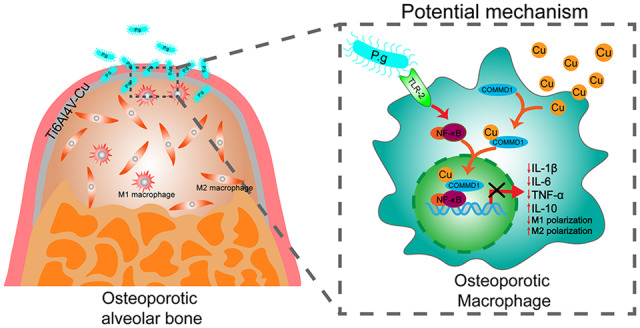
Graphical model of osteoporotic macrophage responses regulated by Cu-bearing Ti6Al4V mesh in infectious microenvironments after GBR surgery.

Taken together, this study demonstrated that the Cu-bearing Ti6Al4V alloy fabricated by SLM can alleviate the over-reaction of osteoporotic macrophages in *P.g*-LPS-induced infectious microenvironments, potentially *via* COMMD1-repressed NF-κB activation (Fig. 9). The decreased M1 macrophage polarization and pro-inflammatory cytokine secretion might impede self-tissue destruction, and the increased M2 macrophage polarization and anti-inflammatory cytokine production favour tissue regeneration in infectious microenvironments. Therefore, it is concluded that Cu-bearing Ti6Al4V alloy can result in ameliorated osteoporotic macrophage responses for the creation of a favourable microenvironment in infectious microenvironments, which holds promise for the development of a GBR-barrier membrane to allow the regeneration of the alveolar bone of osteoporosis patients.

## Supplementary data

Supplementary data are available at *REGBIO* online.

## Funding

This study was supported by National Natural Science Foundation of China (Grant No. 81870766, 51801198), the Science Foundation of Fujian Province (Grant No. 2017J01805), Joint Funds for the Innovation of Sciences and Technology, Fujian Province (Grant No. 2016Y9023) and Startup Fund for scientific research, Fujian Medical University (Grant No. 2019QH2041). 

*Conflict of interest statement*. None declared. 

## Supplementary Material

rbaa045_Supplementary_Data
