# Supplementary material for: Tuning osteoporotic macrophage responses to favour regeneration by Cu-bearing titanium alloy in Porphyromonas gingivalis lipopolysaccharide-induced microenvironments
Source: Regen Biomater. 2020 Dec 3;8(1):rbaa045. doi: 10.1093/rb/rbaa045 (PMC7947590; doi:10.1093/rb/rbaa045)
Supplement: rbaa045_Supplementary_Data [file rbaa045_supplementary_data.docx]

Supplementary Information

Tuning Osteoporotic Macrophage Responses to Favor Regeneration by Cu-bearing Titanium Alloy in *Porphyromonas gingivalis* Lipopolysaccharide-induced Microenvironments

Xiongcheng Xu^a,c^, Yanjin Lu^b,d,*^, Ling Zhou^e^, Mengjiao He^a,c^, Jin Zhuo^a,c^, Quan Zhong^a^, Kai Luo^a,c,*^ and Jinxin Lin^b,d,*^

^a^ Fujian Key Laboratory of Oral Diseases & Fujian Provincial Engineering Research Center of Oral Biomaterial & Stomatological Key laboratory of Fujian College and University, School and Hospital of Stomatology, Fujian Medical University, Fuzhou 350002, China

^b^ Key Laboratory of Optoelectronic Materials Chemistry and Physics, Fujian Institute of Research on the Structure of Matter, Chinese Academy of Sciences, 155 West Yangqiao Road, Fuzhou 350002, China

^c^ Institute of Stomatology & Laboratory of Oral Tissue Engineering, School and Hospital of Stomatology, Fujian Medical University, Fuzhou 350002, China

^d^ University of Chinese Academy of Sciences, 19 Yuquan Road, Beijing 1000049, China

^e^ Department of Stomatology, Fujian Provincial Governmental Hospital & Fujian Health College Affiliated Hospital, Fuzhou 350003, China

* Corresponding author: Kai Luo, #246 Yangqiao Zhong Rd, Fuzhou, Fujian 350002, China.

Yanjin Lu, #155 West Yangqiao Rd, Fuzhou, Fujian 350002, China.

Jinxin Lin, #155 West Yangqiao Rd, Fuzhou, Fujian 350002, China.

E-mail addresses: luokai39@163.com (Kai Luo), yjlu@fjirsm.ac.cn (Yanjin Lu), franklin@fjirsm.ac.cn (Jinxin Lin).

- Supplementary Figure S.1


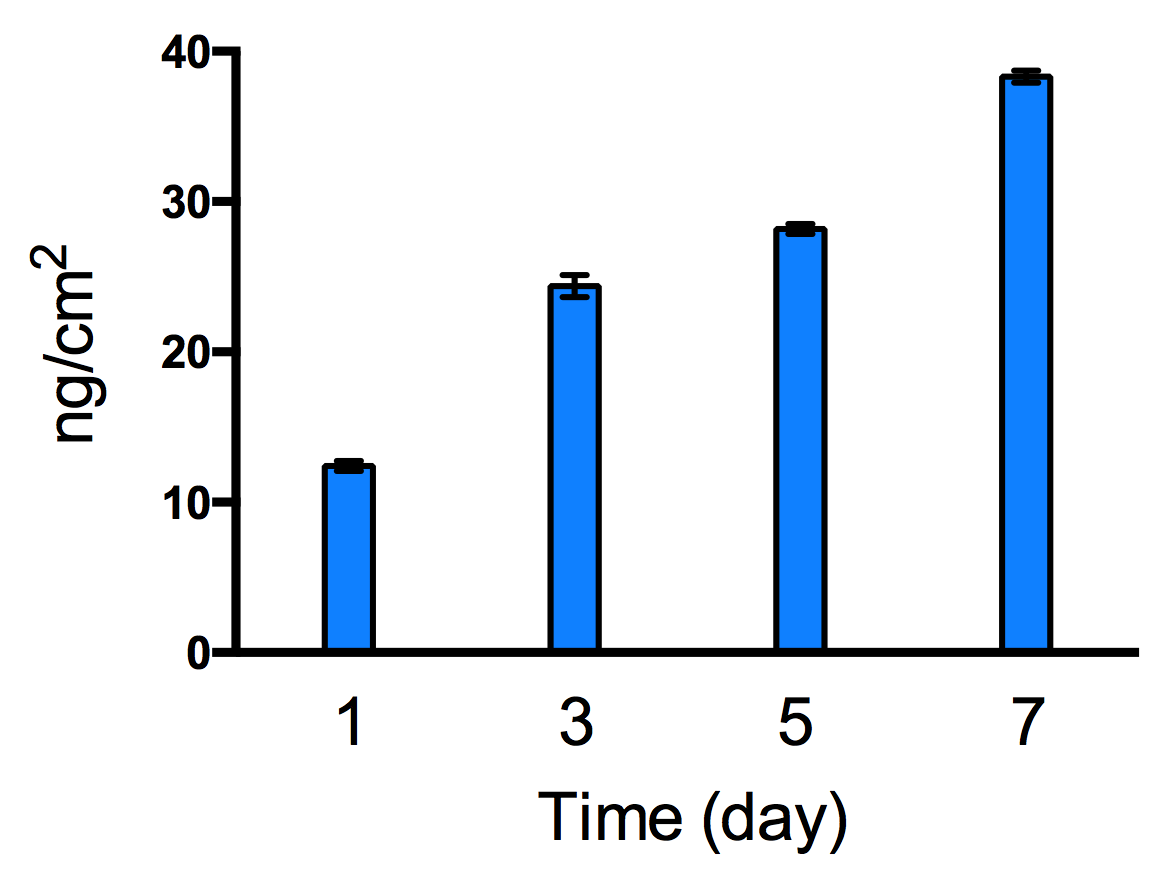


Figure S.1. Amount of Copper Ions Released from the Ti6Al4V−Cu Alloy Immersed in PBS.
